# Probabilistic ancestry maps: a method to assess and visualize population substructures in genetics

**DOI:** 10.1186/s12859-019-2680-1

**Published:** 2019-03-07

**Authors:** Héléna A. Gaspar, Gerome Breen

**Affiliations:** 1 King’s College London; Institute of Psychiatry, Psychology and Neuroscience; Social, Genetic and Developmental Psychiatry (SGDP) Centre, 16 De Crespigny Park, London, SE5 8AF UK; 2 National Institute for Health Research Biomedical Research Centre; South London and Maudsley National Health Service Trust, London, UK

**Keywords:** Generative topographic mapping, Ancestry, Genetics, Population stratification

## Abstract

**Background:**

Principal component analysis (PCA) is a standard method to correct for population stratification in ancestry-specific genome-wide association studies (GWASs) and is used to cluster individuals by ancestry. Using the 1000 genomes project data, we examine how non-linear dimensionality reduction methods such as t-distributed stochastic neighbor embedding (t-SNE) or generative topographic mapping (GTM) can be used to provide improved ancestry maps by accounting for a higher percentage of explained variance in ancestry, and how they can help to estimate the number of principal components necessary to account for population stratification. GTM generates posterior probabilities of class membership which can be used to assess the probability of an individual to belong to a given population - as opposed to t-SNE, GTM can be used for both clustering and classification.

**Results:**

PCA only partially identifies population clusters and does not separate most populations within a given continent, such as Japanese and Han Chinese in East Asia, or Mende and Yoruba in Africa. t-SNE and GTM, taking into account more data variance, can identify more fine-grained population clusters. GTM can be used to build probabilistic classification models, and is as efficient as support vector machine (SVM) for classifying 1000 Genomes Project populations.

**Conclusion:**

The main interest of probabilistic GTM maps is to attain two objectives with only one map: provide a better visualization that separates populations efficiently, and infer genetic ancestry for individuals or populations. This paper is a first application of GTM for ancestry classification models. Our code (https://github.com/hagax8/ancestry_viz) and interactive visualizations (https://lovingscience.com/ancestries) are available online.

**Electronic supplementary material:**

The online version of this article (10.1186/s12859-019-2680-1) contains supplementary material, which is available to authorized users.

## Background

As of 2018, most genome-wide association studies (GWASs) have used populations of European ancestry. However, larger sample sizes are now available and both societal need and funders are mandating more studies focused on other populations. Visualizing and accurately defining complex population structure is therefore of paramount importance. In this paper, we have three aims: to find a better way to visualize population substructures, to define a new procedure to estimate the optimal number of principal components accounting for population stratification, and to obtain an ancestry classification algorithm which can also estimate probabilities to belong to different ancestry groups. This paper focuses on global (genome-wide) ancestry rather than local ancestry defined within chromosome segments.

Principal component analysis (PCA) is widely used to investigate population structure in genetics [1], and to account for population stratification in GWASs (cf. EIGENSTRAT software [2]). However, the 2 or 3 principal components used to build a PCA plot generally account for a small percentage of variance explained and lead to a simplified visualization of population substructures, focused on major continental ancestry, with only partial sensitivity for the identification of admixed individuals or more complex ancestry. Model-based methods such as STRUCTURE [3] and ADMIXTURE [4] provide maximum likelihood estimations of ancestry based on ancestry proportions and allele frequencies but do not provide the simple 2D maps that can be obtained with PCA, multidimensional scaling (MDS), and other multivariate analysis methods.

A PCA ancestry map is constructed from a genotype matrix **G** of dimension *N*×*D*, where the *N* instances are individuals and the *D* features correspond to genetic variants - typically single nucleotide polymorphisms (SNPs) which are pruned to remove SNPs in high linkage disequilibrium with each other so that the identified principal components do not reflect local haplotype structure, but instead reflect genome-wide ancestry. For example, *G*_*nd*_ could be the minor allele count for SNP *d* in individual *n*. For visualization purposes, PCA is used to map **G** to a more interpretable latent or hidden space of 2 or 3 dimensions: **G**→**X**, where X has dimension *N*×2 or *N*×3. The new variables - typically two for a PCA plot - are the first principal components, which account for the highest percentage of the overall variance. However, the total percentage of variance explained by such a small number of principal components can be low for high-dimensional genotype matrices.

More complex visualization methods such as t-distributed stochastic neighbor embedding (t-SNE) [5] or generative topographic mapping (GTM) [6], which are manifold-based and non-linear dimensionality reduction algorithms, are able to capture more information by embedding a *D*-dimensional space in a low-dimensional latent space, where *D* can be any number of features. Instead of two or three principal components, any number of principal components can be used with these methods. To assess the percentage of variance to account for population substructures, we propose to execute two mappings, first carrying out PCA to select principal components and then using t-SNE or GTM: **G**→**X**^′^→**X**, where **X’** is the matrix of *F* principal components (*F*>2), and **X** is the final t-SNE or GTM projection in a 2-dimensional space. The performance of ancestry classification models built with **X** or the visual assessment of clusters in **X** could then provide a way to estimate the number of principal components to account for population stratification.

Both t-SNE and GTM are used for clustering tasks. However, new instances cannot be projected onto a t-SNE map without training the map once again. GTM, on the other hand, not only allows for the projection of new data points, but comes with a probabilistic framework to build a comprehensive classification model and assign probabilities of class membership. t-SNE is now widely used in genetics, and has already been applied to visual population stratification [7], transcriptome visualization [8], and single-cell analysis [9]. GTM is more popular in cheminformatics, and was used to classify chemical compounds [10] or to compare chemical libraries [11]. GTM could easily be transposed to genetics and used to predict ancestry and relative degree of admixture in an individual or a group.

In this paper, 1000 Genomes Project Phase III [12] data is used to build the genotype matrix **G**. The 1000 Genomes Project has gathered genotypes from 26 different populations corresponding to 5 superpopulations: Africans (AFR), Admixed Americans (AMR), East Asians (EAS), Europeans (EUR) and South Asians (SAS). We separated these populations into a training set of 20 populations, and an external test set of 6 populations: Americans of African ancestry in Southwest USA (ASW); African Caribbeans in Barbados (ACB); Mexican ancestry from Los Angeles USA (MXL); Gujarati Indian from Houston, Texas (GIH); Sri Lankan Tamil from the UK (STU); and Indian Telugu from the UK (ITU). Ancestry maps are investigated to cluster and visualize superpopulations and populations using PCA, t-SNE, and GTM. t-SNE and GTM maps accounting for 3 to 1000 principal components are compared to a simple PCA plot. We also compare GTM ancestry classification models to two different algorithms: k-nearest neighbors (*k*-NN) models based on the 2D PCA plot, and linear Support Vector Machine (SVM), a classical machine learning algorithm [13]. We also demonstrate how to assess probabilities of ancestry membership in individuals and populations using GTM.

## Results

### Classification of 5 superpopulations

**Fig. 1:**
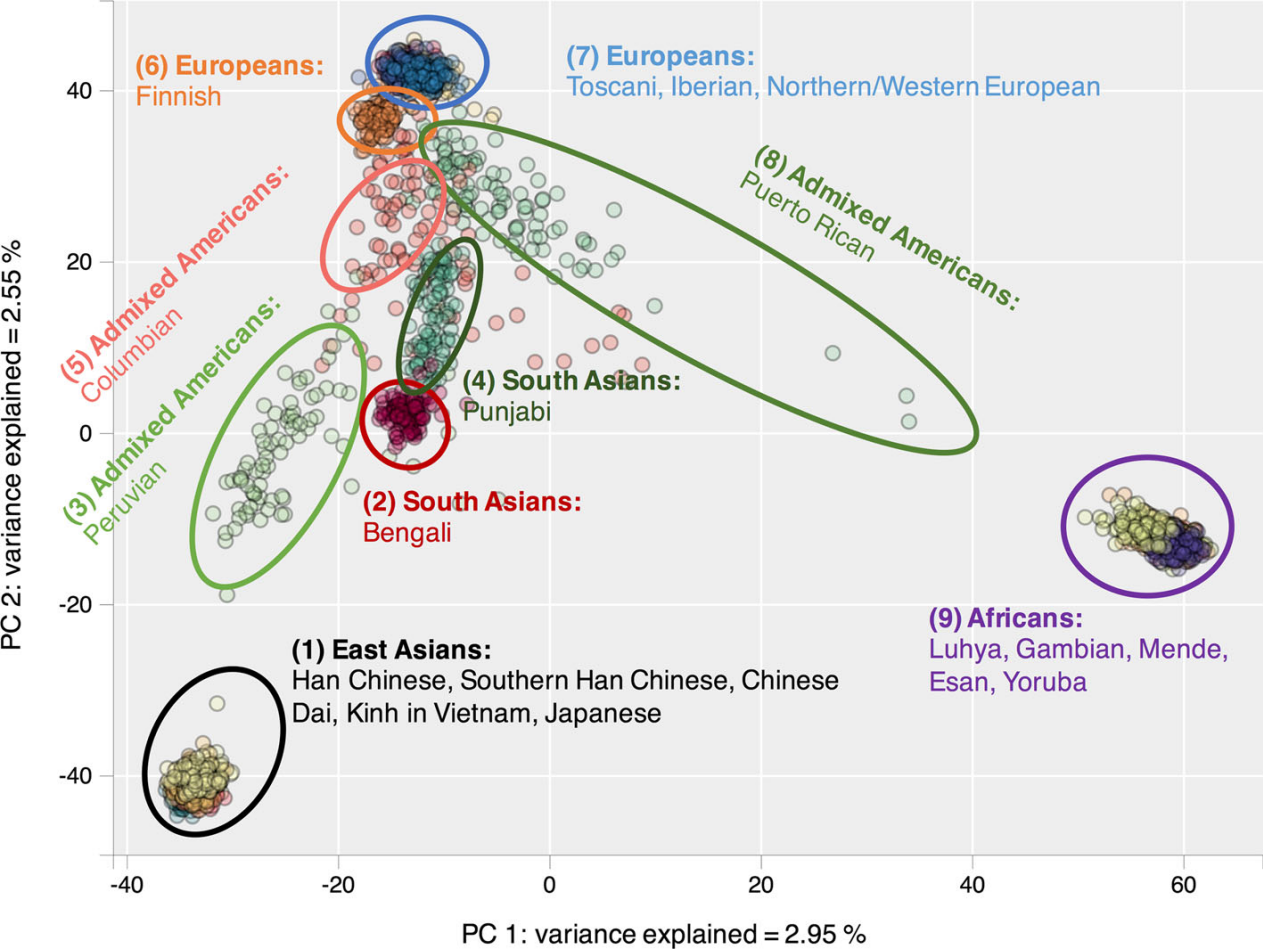
PCA clustering Principal Component Analysis (PCA) plot of 20 populations from 1000 Genomes Project, built using 2 first principal components. The following populations were not used to build the map: ASW = Americans of African Ancestry in SW USA; ACB = African Caribbeans in Barbados; MXL = Mexican Ancestry from Los Angeles USA; GIH = Gujarati Indian from Houston, Texas; STU = Sri Lankan Tamil from the UK; ITU = Indian Telugu from the UK

Visualizations and complete model performance statistics can be found in Additional files 1, 2, 3, 4, 5, 6, 7, 8, 9, 10, 11, 12, 13, 14. PCA clusters and predicts the 5 superpopulations in 1000 Genomes Project efficiently (F1 score = 0.98, cf. Table 1 and Fig. 1): Europeans, Africans, South Asians, East Asians, and Admixed Americans. However, SVM and GTM models with 3 or 10 principal components have higher recall for Admixed Americans and higher precision for South Asians (cf. Additional files 13 and 14). Optimal performances can be achieved by including a third principal component.

**Table 1:** 10 times repeated 5-fold cross-validated F1 score in five 1000 Genomes Project superpopulations using SVM, PCA or GTM

Ancestry	1000G code	PCA 8-NN	SVM 10 PCs	GTM 3 PCs	GTM 10 PCs
Africans	AFR	1.00±0.00	1.00±0.00	1.00±0.00	1.00±0.00
Admixed Americans	AMR	0.93±0.00	1.00±0.00	1.00±0.00	1.00±0.00
East Asians	EAS	1.00±0.00	1.00±0.00	1.00±0.00	1.00±0.00
Europeans	EUR	0.99±0.00	1.00±0.00	1.00±0.00	1.00±0.00
South Asians	SAS	0.93±0.01	1.00±0.00	1.00±0.00	1.00±0.00
Overall F1 score		0.98±0.00	1.00±0.00	1.00±0.00	1.00±0.00

SVM10 = support vector machine classification model using 10 principal components, PCA = k-nearest neighbours model based on 2D PCA map (k = 7), GTM{3,10,100} = bayesian classification model based on generative topographic mapping using 3, 10 or 100 principal components. Each value is an average with 95% confidence interval

**Fig. 2:**
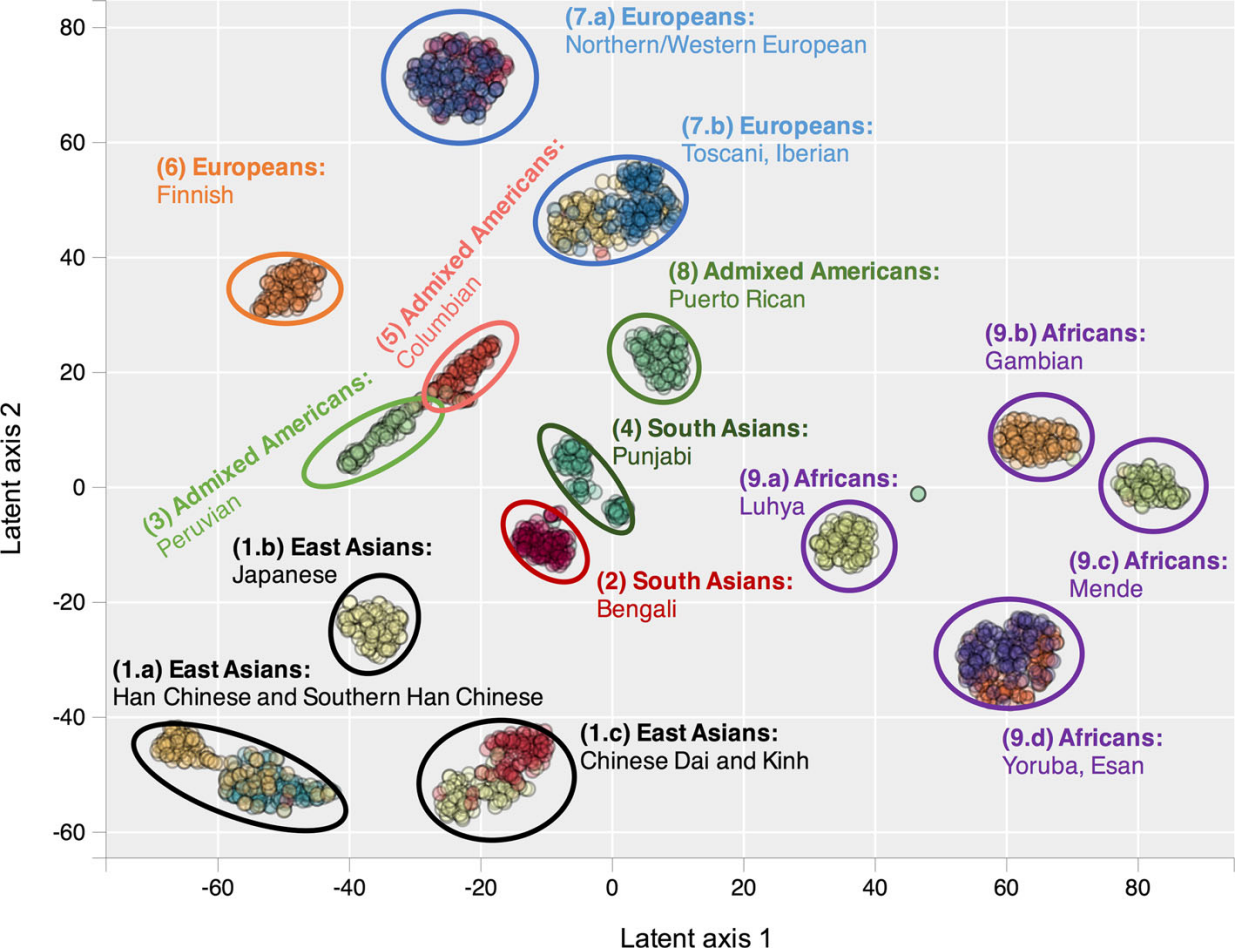
GTM clustering with 10 principal components Generative Topographic Mapping (GTM) plot of 20 populations from 1000 Genomes Project, built using 10 first principal components. The following populations were not used to build the map: ASW = Americans of African Ancestry in SW USA; ACB = African Caribbeans in Barbados; MXL = Mexican Ancestry from Los Angeles USA; GIH = Gujarati Indian from Houston, Texas; STU = Sri Lankan Tamil from the UK; ITU = Indian Telugu from the UK

**Fig. 3:**
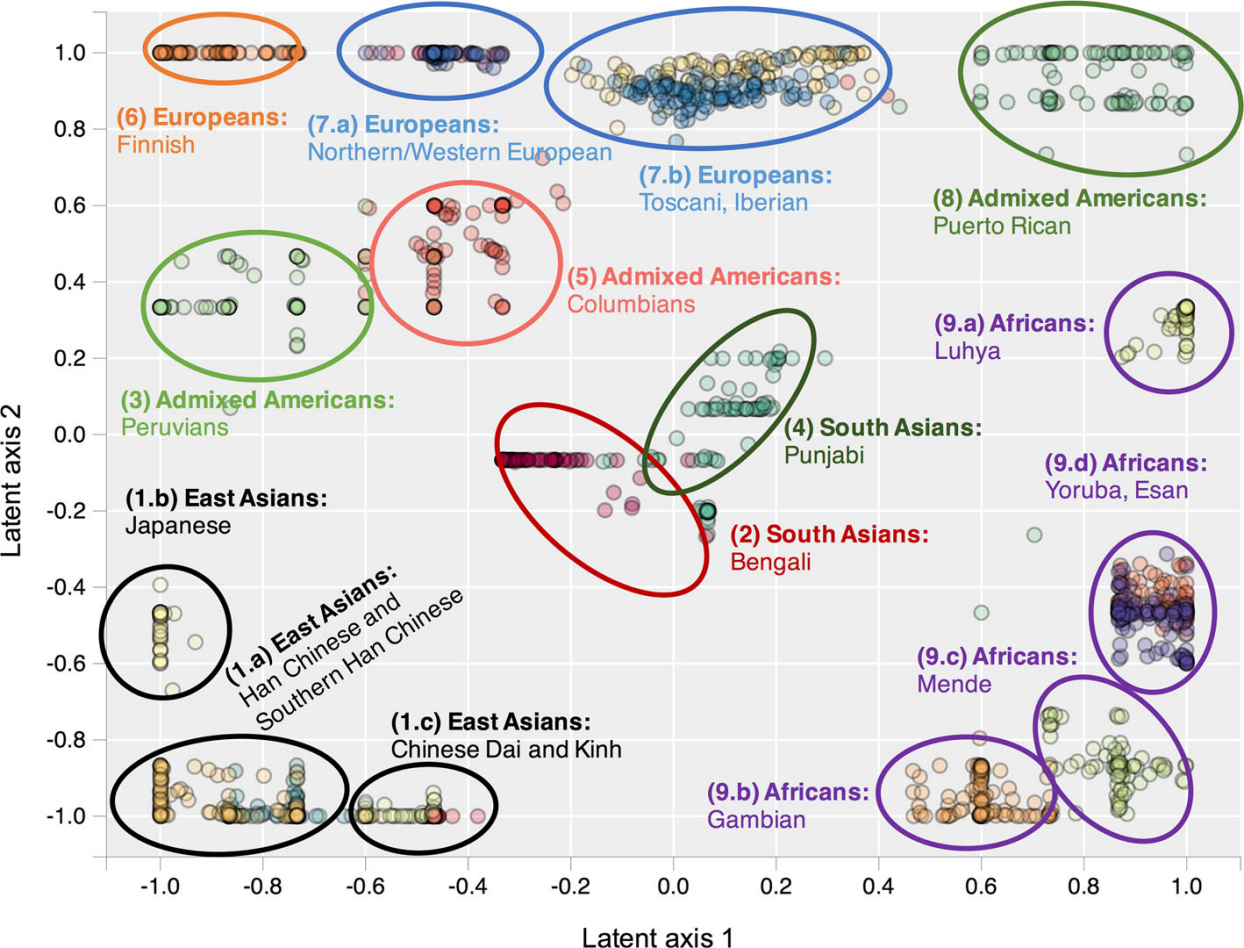
t-SNE clustering with 10 principal components t-distributed stochastic neighbor embedding (t-SNE) plot of 20 populations from 1000 Genomes Project, built using 10 first principal components. The following populations were not used to build the map: ASW = Americans of African Ancestry in SW USA; ACB = African Caribbeans in Barbados; MXL = Mexican Ancestry from Los Angeles USA; GIH = Gujarati Indian from Houston, Texas; STU = Sri Lankan Tamil from the UK; ITU = Indian Telugu from the UK

From Figs. 2 and 3, it can be seen that t-SNE and GTM recognize the same clusters. However, GTM suffers from a packing effect, which results in data points being packed together on a map. t-SNE remedies this situation with Student’s t-distributions in the latent space, which allow small distances between data points in the original space to be translated into larger distances in the 2D latent space.

### Classification performances for 19 ancestry classes

In Table 2, we report performance measures (10 times repeated 5-fold cross-validated F1 score) for SVM, GTM with 3 or 10 principal components, and PCA classification models based on 19 ancestry classes (CEU and GBR populations were merged) from 1000 Genomes Project. Although the PCA plot performs rather well for the 5 classes problem, it cannot properly classify the 19 finer population classes - except for Finnish (FIN), Puerto Ricans (PUR), Peruvians (PEL), Punjabi (PJL) and Bengali (BEB). On the other hand, GTM and SVM models built from only 10 principal components can efficiently classify individuals from most of the 1000 Genomes Project populations (F1 score = 0.80). Some populations are never properly separated, even in sophisticated models taking into account more principal components; this indicates that these populations have a high genetic overlap. This is the case between the Chinese Dai (CDX) and the Kinh in Vietnam (KHV), between the Yoruba (YRI) and Esan (ESN) populations in Nigeria, and between Toscani (TSI) and Iberian populations (IBS) in Europe.

**Table 2:** 10 times repeated 5-fold cross-validated F1 score for 19 population classes from 1000 Genomes Project using SVM, PCA or GTM

Ancestry	1000G code	Population	PCA 8-NN	SVM 10 PCs	GTM 3 PCs	GTM 10 PCs
EAS	CHB	Han Chinese	0.20±0.01	0.78±0.01	0.45±0.04	0.75±0.01
EAS	JPT	Japanese	0.37±0.02	1.00±0.00	0.80±0.01	1.00±0.00
EAS	CHS	Southern Han Chinese	0.34±0.02	0.80±0.01	0.54±0.02	0.80±0.01
EAS	CDX	Chinese Dai	0.24±0.02	0.10±0.02	0.51±0.03	0.44±0.08
EAS	KHV	Kinh in Vietnam	0.44±0.01	0.68±0.00	0.63±0.01	0.71±0.01
EUR	CEU+GBR	Northern/Western Eur.	0.75±0.01	0.99±0.00	0.79±0.01	0.99±0.00
EUR	TSI	Toscani	0.46±0.01	0.74±0.02	0.58±0.01	0.54±0.06
EUR	FIN	Finnish	0.95±0.01	0.99±0.00	0.91±0.01	0.99±0.01
EUR	IBS	Iberian	0.35±0.03	0.81±0.01	0.35±0.04	0.74±0.02
AFR	YRI	Yoruba in Nigeria	0.30±0.02	0.69±0.00	0.15±0.03	0.66±0.03
AFR	LWK	Luhya	0.67±0.01	1.00±0.00	0.59±0.01	1.00±0.00
AFR	GWD	Gambian	0.26±0.02	0.94±0.02	0.23±0.02	0.78±0.07
AFR	MSL	Mende	0.25±0.03	0.93±0.02	0.35±0.03	0.81±0.04
AFR	ESN	Esan in Nigeria	0.28±0.02	0.00±0.01	0.19±0.05	0.28±0.13
AMR	PUR	Puerto Ricans	0.90±0.01	0.86±0.02	0.90±0.01	0.87±0.03
AMR	CLM	Colombians	0.69±0.01	0.85±0.01	0.84±0.01	0.82±0.02
AMR	PEL	Peruvians	0.88±0.01	0.97±0.01	0.94±0.01	0.95±0.01
SAS	PJL	Punjabi	0.89±0.01	0.96±0.01	0.96±0.01	0.96±0.00
SAS	BEB	Bengali	0.95±0.01	0.96±0.01	0.96±0.01	0.96±0.01
Overall			0.54±0.00	0.80±0.00	0.61±0.01	0.80±0.01

SVM 10 PCs = support vector machine classification model using 10 principal components, PCA 8-NN = k-nearest neighbours model based on 2D PCA map (k = 8), GTM 3 or 10 PCs = bayesian classification model based on generative topographic mapping using 3 or 10 principal components. Ancestry codes: EAS = East Asians, EUR = Europeans, AFR = Africans, AMR = Admixed Americans, SAS = South Asians. CEU and GBR were merged into one class. Each value is an average with 95% confidence interval

**Fig. 4:**
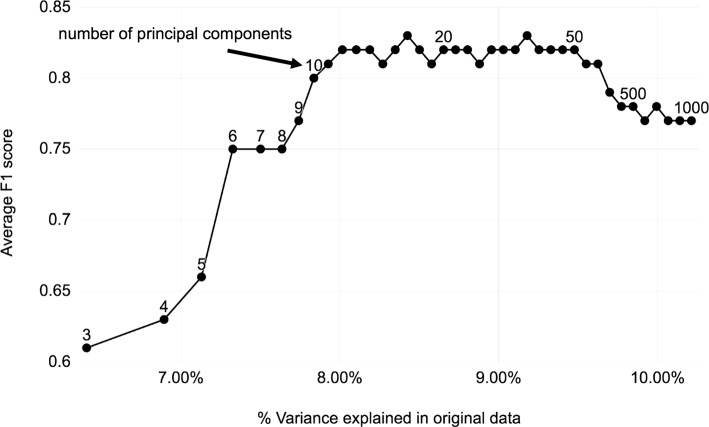
Ancestry classification performance vs. variance explained Generative Topographic Mapping (GTM) ancestry classification model performance as a function of number of principal components used to train the model

To investigate how the performance of 19 populations classification models (with CEU and GBR populations merged into one class) is changing depending on the percentage of variance explained, the cross-validated performance of GTM maps was evaluated by varying the number of principal components included in the model (Fig. 4). The F1 score increases until it reaches a plateau around 0.80 at 10-12 principal components accounting for around 8% variance explained. Interestingly, beyond 100-200 principal components the performance starts decreasing. This could be due to including more individual-level variance, which would disperse population clusters, or to the curse of dimensionality, which occurs when the number of variables increases but not enough data points are provided to populate the high-dimensional space. This indicates that the number of principal components should be optimized - our curve suggests to use 10-12 components for this pruned genotype matrix.

**Fig. 5:**
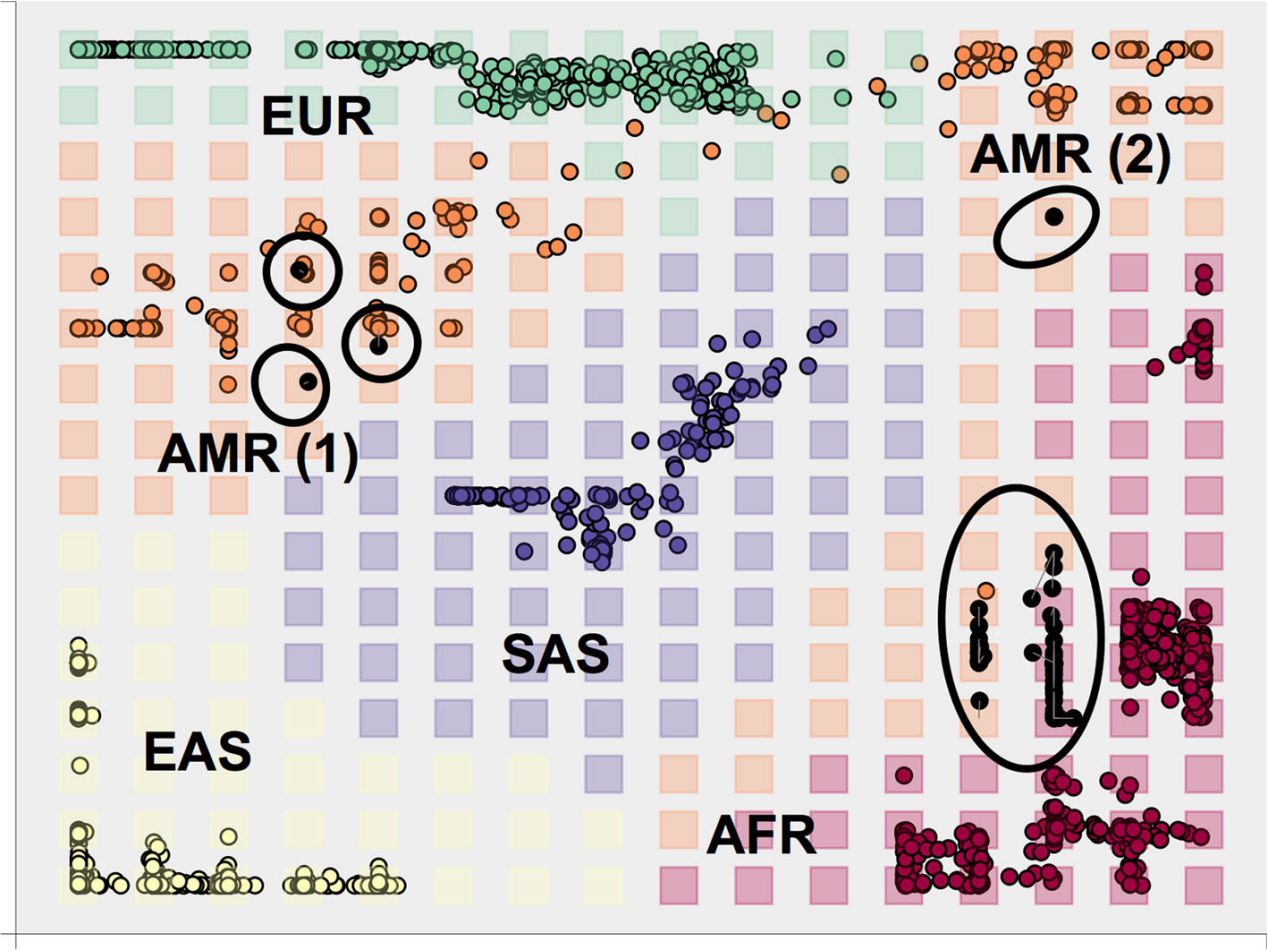
Projected Americans of African ancestry in Southwest USA (ASW) on a GTM map. Generative Topographic Map (GTM) trained with 10 principal components. Coloured points represent individuals coloured by ancestry or superpopulation (AFR, AMR, EAS, EUR, SAS). Squares represent GTM nodes coloured by most probable ancestry. The highlighted black points represent mean positions of ASW individuals projected onto the map. Grey lines map mean positions of individuals on the map to their most probable node. Ancestry codes: EAS = East Asians, EUR = Europeans, AFR = Africans, AMR = Admixed Americans, SAS = South Asians

A final map was built with 10 principal components and the complete training set of 20 populations (cf. Fig. 5). The six populations that were not used to build the GTM map were used to generate posterior probabilities of superpopulation membership, which can be interpreted as the probability for a tested population *pop* to belong to a superpopulation: *P*(*AFR*|*pop*) would be the probability of African ancestry for tested population *pop*. Results are presented in Table 3. Indian Telugu from the UK (ITU), Sri Lankan Tamil from the UK (STU), and Gujarati Indian from Houston (GIH) are all predicted as South Asians with *P*(*SAS*|*pop*)=1 - none of them is mapped to another ancestry group. Individuals with Mexican ancestry from Los Angeles (MXL) are mostly mapped as Admixed Americans with a small European membership probability, whereas Americans of African ancestry in Southwest USA (ASW) and African Caribbeans in Barbados (ACB) show more mixed results - with high probabilities for both African and Admixed American superpopulations. Figure 5 shows how Americans of African ancestry in Southwest USA are distributed on the map: most of them are mapped near the African ancestry group but are assigned to empty nodes, where no African individual in the training set was mapped; some others are close to the Colombian/Peruvian group (AMR 1) and others to the Puerto Rican group (AMR 2).

**Table 3:** Posterior probabilities of superpopulation memberships in 6 test populations obtained by a GTM model trained with all superpopulations

Population (pop)	*P*(*AFR*|*pop*)	*P*(*AMR*|*pop*)	*P*(*EAS*|*pop*)	*P*(*EUR*|*pop*)	*P*(*SAS*|*pop*)
ASW	0.55	0.45	0	0	0
ACB	0.89	0.11	0	0	0
MXL	0	0.98	0	0.02	0
GIH	0	0	0	0	1
STU	0	0	0	0	1
ITU	0	0	0	0	1

NB: GTM classification models are restricted by an applicability domain defined by the training set. Here, the training set contains twenty 1000 Genomes Project, excluding [ASW, ACB, MXL, GIH, STU, ITU]. These posterior probabilities should be considered as a similarity measure between test populations and populations used to build the map, and not as an absolute measure of population admixture. Abbreviations: ASW = Americans of African Ancestry in SW USA; ACB = African Caribbeans in Barbados; MXL = Mexican Ancestry from Los Angeles USA; GIH = Gujarati Indian from Houston, Texas; STU = Sri Lankan Tamil from the UK; ITU = Indian Telugu from the UK; EUR = Europeans; EAS = East Asians; AMR = Admixed Americans; SAS = South Asians

### Additional analysis 1: African-only GTM

A separate GTM was built with African populations exclusively (cf. Additional file 15). Americans of African ancestry in Southwest USA (ASW) and Africans Caribbeans in Barbados (ACB) were excluded from the training set, which included: Esan in Nigeria (ESN); Yoruba in Ibadan, Nigeria (YRI); Gambian in Western Divisions in The Gambia (GWD); Luhya in Webuye, Kenya (LWK); and Mende in Sierra Leone (MSL). We projected onto this African-only map ASW and ACB populations, but also other superpopulations (EUR, EAS, SAS, AMR), in order to distinguish populations based on their African variation. ASW and ACB are both mapped near Nigerian populations, whereas all other superpopulations (EUR, EAS, SAS, and AMR) are mapped in the same approximate location near the Luhya (LWK) - posterior probabilities of ancestry membership are provided in Table 4. However, these superpopulations are mapped in locations that are not populated by the training set; no strong conclusion should be inferred from these results. Moreover, the 1000 Genomes Project does not contain many African ethnic groups. Constructing an African-only map with more ethnic groups would be an interesting follow-up to this analysis.

**Table 4:** Posterior probabilities of African ethnicity membership in 6 test populations obtained by a GTM model trained on African populations exclusively

Population (pop)	*P*(*ESN*|*pop*)	*P*(*YRI*|*pop*)	*P*(*GWD*|*pop*)	*P*(*LWK*|*pop*)	*P*(*MSL*|*pop*)
ASW	0.24	0.37	0.11	0.13	0.14
ACB	0.29	0.42	0.07	0.07	0.15
EUR	0.04	0.10	0.21	0.62	0.04
EAS	0.09	0.19	0.21	0.44	0.07
AMR	0.07	0.15	0.23	0.49	0.06
SAS	0.06	0.13	0.21	0.53	0.05

NB: GTM classification models are restricted by an applicability domain defined by the training set. Here, the training set contains only African populations, excluding ASW and ACB subsets. These posterior probabilities should be considered as a similarity measure between test populations and populations used to build the map, and not as an absolute measure of population admixture. Abbreviations: ASW = Americans of African Ancestry in SW USA; ACB = African Caribbeans in Barbados; ESN = Esan in Nigeria; YRI = Yoruba in Ibadan; Nigeria; GWD = Gambian in Western Divisions in the Gambia; LWK = Luhya in Webuye, Kenya; MSL = Mende in Sierra Leone; EUR = Europeans; EAS = East Asians; AMR = Admixed Americans; SAS = South Asians

### Additional analysis 2: Arabidopsis thaliana

To test our methods on non-human genomes, we generated GTM, t-SNE and PCA maps for 1135 Arabidopsis thaliana genomes (a model plant organism) from the 1001 Genomes Consortium [14]. Visualizations are available in Additional files 16 and 17. PCA can separate the strains by continent but not by individual countries, as opposed to GTM and t-SNE, which find more fine-grained clusters corresponding to individual countries or regions, such as Spain, Southern Sweden, Northern Sweden, Southern Italy, or Northern Italy.

## Discussion

### Defining the training set

Our classification models were trained using known ancestry labels and a reference population (1000 Genomes Project). However, any other reference population could be used as a training set. In this application, populations expected to be more homogeneous were included in the training set. The choice of training set populations could also depend on the goal of the study, such as distinguishing between African populations in an African-only dataset, in which case a better classification model could be built using exclusively African samples.

### Testing new data

To predict the ancestry of new individuals (test set) using a model trained on a reference population (training set), SNPs in the test matrix should correspond to the SNPs in the train matrix. This was not an issue in this paper, where populations from 1000 Genomes Project were used for both training and test. But in the more general case, many of the SNPs in the training set will be missing from the test set. Missing values in the test matrix should be imputed using the reference population, which can be achieved using genome imputation softwares such as MaCH [15] or IMPUTE2 [16].

### Outliers

GTM or t-SNE maps can also be used to identify ancestry outliers, i.e. mislabeled individuals. Outliers are typically mapped to single points far away from their expected clusters. These data points should be removed from the training set used to build the classification model. By observing t-SNE and GTM maps, outliers can readily be identified in the 1000 Genomes Project.

### Hyperparameter optimization

One major drawback of GTM and t-SNE is hyperparameter optimization. GTM has at least four hyperparameters to optimize, and t-SNE at least three. The maps presented in this paper have fixed hyperparameters (cf. Methods). However, hyperparameters might have a significant impact on the shape of the map, and can be optimized to obtain better visualization and classification performance. For GTM classification models, typical performance measures such as the F1 score, balanced accuracy, area under the curve (AUC) or Matthews correlation coefficient (MCC) can be used to select the optimal values for these hyperparameters.

## Conclusion

PCA provides a good visualization of the superpopulations in the 1000 Genomes Project (AFR, AMR, EUR, EAS, SAS), but is not ideal for more fine-grained clustering and does not provide probabilistic models for admixed populations. On the other hand, both t-SNE and GTM provide a way to cluster and visualize more complex population substructures. GTM, as opposed to t-SNE, can be harnessed to generate comprehensive ancestry classification models. Moreover, new individuals can be directly projected onto a pre-constructed GTM map - which makes it the ideal choice to cluster individuals based on pre-defined panels. We showed how to assess ancestry membership probabilities using GTM and interpret them through visualization. By generating t-SNE or GTM maps with increasing number of principal components, we can estimate the percentage of variance explained to identify population substructures - this could also be useful to account for population stratification in genome-wide association studies.

## Methods

### Data and quality control

Genotypes of 2504 people in the 1000 Genomes Project Phase III were downloaded from ftp://ftp.1000genomes.ebi.ac.uk/vol1/ftp/release/20130502 [12]. Variants were removed based on a Hardy-Weinberg equilibrium exact test *p*-value filter (< 0.001) and genotyping rate filter (> 0.02). The Hardy-Weinberg equilibrium test measures whether the ratio between homozygous and heterozygous genotypes differs significantly from prediction under HWE assumptions. SNPs from the major histocompatibility complex (MHC) on chromosome 6 and in the chromosome 8 inversion region were excluded. The remaining SNPs were pruned twice using plink 1.9 [17, 18] with windows of 1000 variants and step size 10, pair-wise squared correlation threshold = 0.02, and minor allele frequency > 0.05. The pruning operation deals with *linkage desequilibrium* or non-random association of alleles at different loci: it reduces the number of SNPs, keeps SNPs in linkage equilibrium, and thereby reduces data dimensionality. A training set was built by removing the following populations: Americans of African ancestry in Southwest USA (code = ASW); African Caribbeans in Barbados (ACB); Mexican ancestry from Los Angeles USA (MXL); Gujarati Indian from Houston, Texas (GIH); Sri Lankan Tamil from the UK (STU); and Indian Telugu from the UK (ITU). We used these populations as an external test set to predict the degree of relative admixture in individuals and populations. For the classification models, we also merged British in England and Scotland (GBR) and Utah Residents with Northern and Western European Ancestry (CEU) to obtain a single category for Northern and Western European Ancestry.

### Additional dataset: Arabidopsis thaliana

We used an additional dataset of 1135 Arabidopsis thaliana genomes extracted from the 1001 Genomes Project [14]; the genotypes and an imputed SNP matrix could be downloaded from 1001genomes.org. Arabidopsis thaliana was the first plant genome to be sequenced and is a commonly used model organism. Variants were removed using a permissive genotyping rate filter (> 0.2). SNPs were pruned using plink 1.9 [17, 18] with windows of 100 variants and step size 10, pair-wise squared correlation threshold = 0.1, and minor allele frequency > 0.05. We merged the imputed SNP matrix with our filtered list of SNPs to obtain a filtered imputed SNP matrix.

### Visualization of ancestry clusters using t-SNE and GTM

t-SNE [5] translates similarities between points into probabilities; Gaussian joint probabilities in the original input space and Student’s t-distributions in the latent space. The Kullback-Leibler divergence between data distributions in the input and latent space is minimized with gradient descent. t-SNE has several parameters to optimize: the learning rate for gradient descent, the perplexity of distributions in the initial space, and the early exaggeration. In this paper, we used the scikit-learn v0.19.1 implementation for t-SNE [19], with default learning rate = 200, perplexity = 30, and early exaggeration = 12. The main disadvantage of t-SNE is its lack of a framework to project new points onto a pre-trained map - a feature available in PCA and GTM.

The core principle of GTM [6] is to fit a manifold into the high-dimensional initial space. The points **y**_*k*_ on the manifold **Y** in the initial space are the centers of normal probability distributions of **g**, which here are individuals described by the genotype matrix **G**:

1 $$\begin{array}{@{}rcl@{}}  p(\mathbf{g}|\mathbf{x}_{k},\mathbf{W},\beta) = \frac{\beta}{2\pi}^{D/2} \exp{\left(-\frac{\beta}{2}\|\mathbf{y}_{k}-\mathbf{g}\|^{2}\right)} \end{array} $$

where *β* is the common inverse variance of these distributions and **W** is the parameters matrix of the mapping function **y**(**x**;**W**) which maps nodes **x**_*k*_ in the latent space to **y**_*k*_: **y**(**x**_*k*_;**W**)=**W***ϕ*(**x**_*k*_), where *ϕ*(**x**_*k*_) is a set of radial basis functions. **W** and *β* are optimized with an expectation-maximization (EM) algorithm maximizing the overall data likelihood. The responsibility or posterior probability that the individual **g**_*n*_ in the original genotype space is generated from the *k*th node in the latent space is computed using Bayes theorem:

2 $$\begin{array}{@{}rcl@{}}  R_{kn} = p(\mathbf{x}_{k} | \mathbf{g}_{n},\mathbf{W},\beta) = \frac{p(\mathbf{g}_{n}|\mathbf{x}_{k},\mathbf{W},\beta) p(\mathbf{x}_{k})}{{\sum\nolimits}_{k^{\prime}=1}^{K} p(\mathbf{g}_{n}|\mathbf{x}_{k^{\prime}},\mathbf{W},\beta)p(\mathbf{x}_{k^{\prime}})} \end{array} $$

These responsibilities are used to compute the mean position of an individual on the map **x**(**g**_*n*_), by averaging over all nodes on the map:

3 $$\begin{array}{@{}rcl@{}}  \mathbf{x}(\mathbf{g}_{n}) = \sum\limits_{k=1}^{K} \mathbf{x}_{k} R_{kn} \end{array} $$

We used the python package ugtm v1.1.4 [20] for generative topographic mapping, and scripts used for ancestry classification are available online (https://github.com/hagax8/ancestry_viz). GTM has several hyperparameters to tune, which might have a high impact on the shape of the map: the number of radial basis functions, a width factor for these functions, map grid size, and a regularization parameter.

### Ancestry classification models

PCA does not provide a comprehensive framework to build a probabilistic classification model. However, a simple classification model associated with the 2-dimensional plot can be built using the *k*-NN approach in three steps: (1) a PCA plot is constructed from a training set, (2) a test set is projected on the plot, and (3) each test individual is assigned the predominant ancestry amongst its *k* nearest neighbors in the training set. We did not construct *k*-NN models for t-SNE since it is not straightforward to project new points onto a t-SNE map. On the other hand, GTM provides a probabilistic framework which can be used to build classification models and generate class membership probabilities [10]. GTM responsibilities can be seen as feature vectors: they encode individuals depending on their position on the map, which is discretized into a finite number of nodes (positions). They can be used to estimate the probability of a specific ancestry given the position on map, using Bayes’ theorem

4 $$\begin{array}{@{}rcl@{}}  P(a|\mathbf{x}_{k})=\frac{P(\mathbf{x}_{k}|a) \times P(a)}{{\sum\nolimits}_{a} P(\mathbf{x}_{k}|a) \times P(a)} \end{array} $$

where *P*(**x**_*k*_|*a*) is computed as follows:

5 $$\begin{array}{@{}rcl@{}}  P(\mathbf{x}_{k}|a)=\frac{{\sum\nolimits}_{n}{R_{kn}}}{N_{a}} \end{array} $$

where *R*_*kn*_ is the responsibility of node **x**_*k*_ for an individual belonging to population *a*, which counts *N*_*a*_ individuals. It is then possible to predict the ancestry profile *P*(*a*|**g**_*i*_) of a new individual with associated responsibilities {*R*_*ki*_}

6 $$\begin{array}{@{}rcl@{}}  P(a|\mathbf{g}_{i})=\sum\limits_{k} P(a|\mathbf{x}_{k}) \times R_{ki} \end{array} $$

GTM nodes **x**_*k*_ can be represented as points coloured by most probable ancestry *a*_max_ using *P*(*a*_max_|**x**_*k*_). We compared performances of visual classifications (PCA and GTM) with linear support vector machine classification (SVM), a classical machine learning algorithm. Linear SVM is only dependent on *C*, the penalty hyperparameter. Increasing *C* increases the variance of the model and decreases its bias. In this application, classification performance is estimated by the average F1 score over all ancestry classes in a 5-fold cross-validation experiment (5-CV) repeated 10 times. The F1 score is a harmonic mean of precision and recall. For each of the 10 repetitions, labels are predicted for 5 partitions of the data, which are concatenated to obtain predicted values for the entire dataset. From these, F1 scores are computed for each class *a* and repetition *j*. The per-class performance measure is computed across the 10 repetitions:

7 $$\begin{array}{@{}rcl@{}}  {F1 score}_{a} = \frac{\sum\limits_{j=1}^{10}{{F1 score}_{aj}}}{10} \end{array} $$

The overall model performance measure is a weighted average across per-class F1 scores, with weights equal to the number of individuals in the class:

8 $$\begin{array}{@{}rcl@{}}  {F1 score}=\left (\sum\limits_{j=1}^{10}{\frac{\sum\limits_{a}{{F1 score}_{aj} \times N_{a}}}{N_{total}}} \right) \div 10 \end{array} $$

This procedure is performed for each parameter combination and for each algorithm (PCA, GTM, SVM). The best model for each algorithm is defined as having the largest overall F1 score. Only the performance of the best model is reported in the Results section. For PCA, we vary *k* (the number of neighbours) from 1 to 10. For GTM, we set the map grid size (number of nodes) = 16*16, the number of RBFs = 4*4, regularization = 0.1 and rbf width factor = 0.3. For linear SVM, the penalty parameter is set to *C*=2^*r*^ where *r* runs from -5 to 10.

### Posterior probabilities of ancestry membership for whole populations

All our models are trained with only twenty 1000 Genomes Project populations. Six populations are used as an external test set (cf. foregoing section Data and quality control). Posterior probabilities of ancestry membership are estimated for all individuals in these test populations (Eq. 6) based on observed superpopulation distributions (Eq. 5). We also generate probabilities of belonging to a superpopulation for each population as a whole, by replacing individual responsibilities {*R*_*ki*_} in equation 6 by an overall population responsibility {*R*_*kp*_}

9 $$ R_{kp}=\frac{{\sum\nolimits}_{i}{R_{ki}}}{N_{i}}  $$

It should be noted that these responsibilities {*R*_*kp*_} correspond to the averaged distribution of the population on the map, and can be used to compare populations and estimate their diversity.

## Additional files

Additional file 1 GTM map of twenty 1000 Genomes Project populations. Interactive GTM map of twenty 1000 Genomes Project populations. File name: 1000G_GTM_20populations.html. The file can be viewed in a web browser with internet access. (HTML 2416 kb)

Additional file 2 t-SNE map of twenty 1000 Genomes Project populations. Interactive t-SNE map of twenty 1000 Genomes Project populations. File name: 1000G_t-SNE_20populations.html. The file can be viewed in a web browser with internet access. (HTML 589 kb)

Additional file 3 GTM projection, test set 1: Americans of African ancestry in SW USA (ASW). Projection of Americans of African ancestry in SW USA (black points) onto a GTM map trained with 10 principal components. File name: 1000G_GTM_projection_ASW.html. The file can be viewed in a web browser with internet access. (HTML 437 kb)

Additional file 4 GTM projection, test set 2: African Caribbeans in Barbados (ACB). Projection of African Caribbeans in Barbados (black points) onto a GTM map trained with 10 principal components. File name: 1000G_GTM_projection_ACB.html. The file can be viewed in a web browser with internet access. (HTML 471 kb)

Additional file 5 GTM projection, test set 3: Mexican Ancestry from Los Angeles USA (MXL). Projection of individuals of Mexican ancestry from Los Angeles USA (black points) onto a GTM map trained with 10 principal components. File name: 1000G_GTM_projection_MXL.html. The file can be viewed in a web browser with internet access. (HTML 439 kb)

Additional file 6 GTM projection, test set 4: Gujarati Indian from Houston, Texas (GIH). Projection of Gujarati Indian from Houston (black points) onto a GTM map trained with 10 principal components. File name: 1000G_GTM_projection_GIH.html. The file can be viewed in a web browser with internet access. (HTML 483 kb)

Additional file 7 GTM projection, test set 5: Sri Lankan Tamil from the UK (STU). Projection of Sri Lankan Tamil from the UK (black points) onto a GTM map trained with 10 principal components. File name: 1000G_GTM_projection_STU.html. The file can be viewed in a web browser with internet access. (HTML 482 kb)

Additional file 8 GTM projection, test set 6: Indian Telugu from the UK (ITU). Projection of Indian Telugu from the UK (black points) onto a GTM map trained with 10 principal components. File name: 1000G_GTM_projection_ITU.html. The file can be viewed in a web browser with internet access. (HTML 482 kb)

Additional file 9 1000 Genomes Project populations. Table of 1000 Genomes Project populations and superpopulations and the number of individuals in each category. File name: 1000G_populations.html. (HTML 7 kb)

Additional file 10 Variance explained in first principal components of genotype matrix. Variance explained in 100 first principal components of the genotype matrix for twenty 1000 Genomes Projects Populations, which were used as a training set to build our models. File name: varianceExplained.html. (HTML 13 kb)

Additional file 11 5-fold cross-validated precision for twenty 1000 Genomes Project populations (19 classes) using SVM, PCA or GTM. Precision of optimized models for the following algorithms: SVM 10 PCs = support vector machine classification model using 10 principal components, PCA 8-NN = k-nearest neighbours model based on 2D PCA map (k = 8), GTM 3 or 10 PCs = bayesian classification model based on generative topographic mapping using 3 or 10 principal components. File name: precision_crossvalidation_19classes.html. (HTML 7 kb)

Additional file 12 5-fold cross-validated recall for twenty 1000 Genomes Project populations (19 classes) using SVM, PCA or GTM. Recall of optimized models for the following algorithms: SVM 10 PCs = support vector machine classification model using 10 principal components, PCA 8-NN = k-nearest neighbours model based on 2D PCA map (k = 8), GTM 3 or 10 PCs = bayesian classification model based on generative topographic mapping using 3 or 10 principal components. File name: recall_crossvalidation_19classes.html. (HTML 8 kb)

Additional file 13 5-fold cross-validated precision for five 1000 Genomes Project superpopulations (5 classes). Precision of optimized models for the following algorithms: SVM 10 PCs = support vector machine classification model using 10 principal components, PCA 8-NN = k-nearest neighbours model based on 2D PCA map (k = 8), GTM 3 or 10 PCs = bayesian classification model based on generative topographic mapping using 3 or 10 principal components. File name: precision_crossvalidation_5classes.html. (HTML 3 kb)

Additional file 14 5-fold cross-validated recall for five 1000 Genomes Project superpopulations (5 classes). Recall of optimized models for the following algorithms: SVM 10 PCs = support vector machine classification model using 10 principal components, PCA 8-NN = k-nearest neighbours model based on 2D PCA map (k = 8), GTM 3 or 10 PCs = bayesian classification model based on generative topographic mapping using 3 or 10 principal components. File name: recall_crossvalidation_5classes.html. (HTML 3 kb)

Additional file 15 African-only GTM map. Interactive GTM map for AFR superpopulation (1000 Genomes Project), excluding ASW and ACB populations, and projections of following test sets: two African ancestry populations (ASW and ACB), and 1000 Genomes superpopulations (EUR, EAS, AMR, and SAS) on the AFR map).File name: AFR_maps.pdf. (PDF 1414 kb)

Additional file 16 Arabidopsis map coloured by country. Interactive map of 1135 Arabidopsis thaliana genomes from the 1001 Genomes project. File name: worldmap_arabidopsis_countries.html. The file can be viewed in a web browser with internet access. (HTML 571 kb)

Additional file 17 Arabidopsis map coloured by admixture group. Interactive map of 1135 Arabidopsis thaliana genomes from the 1001 Genomes project, coloured by admixture group. File name: worldmap_arabidopsis_admixed.html. The file can be viewed in a web browser with internet access. (HTML 571 kb)

## Abbreviations

AFR: African
AMR: Admixed American
EAS: East Asian
EUR: European
GTM: Generative topographic mapping
GWAS: Genome-wide assocation study
PCA: Principal component analysis
SAS: South Asian
SNP: Single nucleotide polymorphism
SVM: Support vector machine
t-SNE: t-distributed stochastic neighbor embedding
