# Supplementary material for: Probabilistic ancestry maps: a method to assess and visualize population substructures in genetics
Source: BMC Bioinformatics. 2019 Mar 7;20:116. doi: 10.1186/s12859-019-2680-1 (PMC6407257; doi:10.1186/s12859-019-2680-1)
Supplement: Supplementary file 9 — 1000 Genomes Project populations. Table of 1000 Genomes Project populations and superpopulations and the number of individuals in each category. File name: 1000G_populations.html. (HTML 7 kb) [file 12859_2019_2680_MOESM9_ESM.html]

| Superpopulation code | Population code | Population | Included in train set | Sample size |
| --- | --- | --- | --- | --- |
| AFR | ACB | African Caribbeans in Barbados | no | 96 |
| AFR | ASW | Americans of African Ancestry in SW USA | no | 61 |
| SAS | BEB | Bengali from Bangladesh | yes | 86 |
| EAS | CDX | Chinese Dai in Xishuangbanna, China | yes | 93 |
| EUR | CEU | Utah Residents (CEPH) with Northern and Western European Ancestry | yes | 99 |
| EAS | CHB | Han Chinese in Beijing, China | yes | 103 |
| EAS | CHS | Southern Han Chinese | yes | 105 |
| AMR | CLM | Colombians from Medellin, Colombia | yes | 94 |
| AFR | ESN | Esan in Nigeria | yes | 99 |
| EUR | FIN | Finnish in Finland | yes | 99 |
| EUR | GBR | British in England and Scotland | yes | 91 |
| SAS | GIH | Gujarati Indian from Houston, Texas | no | 103 |
| AFR | GWD | Gambian in Western Divisions in the Gambia | yes | 113 |
| EUR | IBS | Iberian Population in Spain | yes | 107 |
| SAS | ITU | Indian Telugu from the UK | no | 102 |
| EAS | JPT | Japanese in Tokyo, Japan | yes | 104 |
| EAS | KHV | Kinh in Ho Chi Minh City, Vietnam | yes | 99 |
| AFR | LWK | Luhya in Webuye, Kenya | yes | 99 |
| AFR | MSL | Mende in Sierra Leone | yes | 85 |
| AMR | MXL | Mexican Ancestry from Los Angeles USA | no | 64 |
| AMR | PEL | Peruvians from Lima, Peru | yes | 85 |
| SAS | PJL | Punjabi from Lahore, Pakistan | yes | 96 |
| AMR | PUR | Puerto Ricans from Puerto Rico | yes | 104 |
| SAS | STU | Sri Lankan Tamil from the UK | no | 102 |
| EUR | TSI | Toscani in Italia | yes | 107 |
| AFR | YRI | Yoruba in Ibadan, Nigeria | yes | 108 |
