# Supplementary material for: Probabilistic ancestry maps: a method to assess and visualize population substructures in genetics
Source: BMC Bioinformatics. 2019 Mar 7;20:116. doi: 10.1186/s12859-019-2680-1 (PMC6407257; doi:10.1186/s12859-019-2680-1)
Supplement: Supplementary file 10 — Variance explained in first principal components of genotype matrix. Variance explained in 100 first principal components of the genotype matrix for twenty 1000 Genomes Projects Populations, which were used as a training set to build our models. File name: varianceExplained.html. (HTML 13 kb) [file 12859_2019_2680_MOESM10_ESM.html]

| n = Principal Component (PC) | Variance explained by the nth PC | % Variance explained by n first PCs |
| --- | --- | --- |
| 1 | 2.95E-02 | 2.95% |
| 2 | 2.55E-02 | 5.50% |
| 3 | 9.04E-03 | 6.41% |
| 4 | 4.88E-03 | 6.89% |
| 5 | 2.35E-03 | 7.13% |
| 6 | 1.97E-03 | 7.33% |
| 7 | 1.75E-03 | 7.50% |
| 8 | 1.35E-03 | 7.64% |
| 9 | 1.05E-03 | 7.74% |
| 10 | 9.52E-04 | 7.84% |
| 11 | 9.07E-04 | 7.93% |
| 12 | 8.89E-04 | 8.02% |
| 13 | 8.74E-04 | 8.10% |
| 14 | 8.51E-04 | 8.19% |
| 15 | 8.19E-04 | 8.27% |
| 16 | 7.84E-04 | 8.35% |
| 17 | 7.67E-04 | 8.43% |
| 18 | 7.60E-04 | 8.50% |
| 19 | 7.59E-04 | 8.58% |
| 20 | 7.57E-04 | 8.65% |
| 21 | 7.55E-04 | 8.73% |
| 22 | 7.54E-04 | 8.80% |
| 23 | 7.52E-04 | 8.88% |
| 24 | 7.51E-04 | 8.95% |
| 25 | 7.50E-04 | 9.03% |
| 26 | 7.49E-04 | 9.10% |
| 27 | 7.48E-04 | 9.18% |
| 28 | 7.46E-04 | 9.25% |
| 29 | 7.45E-04 | 9.33% |
| 30 | 7.44E-04 | 9.40% |
| 31 | 7.42E-04 | 9.48% |
| 32 | 7.41E-04 | 9.55% |
| 33 | 7.40E-04 | 9.62% |
| 34 | 7.39E-04 | 9.70% |
| 35 | 7.38E-04 | 9.77% |
| 36 | 7.37E-04 | 9.85% |
| 37 | 7.36E-04 | 9.92% |
| 38 | 7.35E-04 | 9.99% |
| 39 | 7.34E-04 | 10.07% |
| 40 | 7.34E-04 | 10.14% |
| 41 | 7.33E-04 | 10.21% |
| 42 | 7.32E-04 | 10.29% |
| 43 | 7.31E-04 | 10.36% |
| 44 | 7.30E-04 | 10.43% |
| 45 | 7.29E-04 | 10.51% |
| 46 | 7.29E-04 | 10.58% |
| 47 | 7.28E-04 | 10.65% |
| 48 | 7.27E-04 | 10.72% |
| 49 | 7.27E-04 | 10.80% |
| 50 | 7.26E-04 | 10.87% |
| 51 | 7.25E-04 | 10.94% |
| 52 | 7.24E-04 | 11.01% |
| 53 | 7.23E-04 | 11.09% |
| 54 | 7.23E-04 | 11.16% |
| 55 | 7.22E-04 | 11.23% |
| 56 | 7.22E-04 | 11.30% |
| 57 | 7.21E-04 | 11.38% |
| 58 | 7.20E-04 | 11.45% |
| 59 | 7.20E-04 | 11.52% |
| 60 | 7.20E-04 | 11.59% |
| 61 | 7.19E-04 | 11.66% |
| 62 | 7.18E-04 | 11.74% |
| 63 | 7.17E-04 | 11.81% |
| 64 | 7.17E-04 | 11.88% |
| 65 | 7.16E-04 | 11.95% |
| 66 | 7.15E-04 | 12.02% |
| 67 | 7.15E-04 | 12.09% |
| 68 | 7.14E-04 | 12.16% |
| 69 | 7.13E-04 | 12.24% |
| 70 | 7.12E-04 | 12.31% |
| 71 | 7.11E-04 | 12.38% |
| 72 | 7.11E-04 | 12.45% |
| 73 | 7.11E-04 | 12.52% |
| 74 | 7.10E-04 | 12.59% |
| 75 | 7.09E-04 | 12.66% |
| 76 | 7.09E-04 | 12.73% |
| 77 | 7.08E-04 | 12.80% |
| 78 | 7.07E-04 | 12.87% |
| 79 | 7.07E-04 | 12.95% |
| 80 | 7.07E-04 | 13.02% |
| 81 | 7.06E-04 | 13.09% |
| 82 | 7.06E-04 | 13.16% |
| 83 | 7.05E-04 | 13.23% |
| 84 | 7.05E-04 | 13.30% |
| 85 | 7.04E-04 | 13.37% |
| 86 | 7.04E-04 | 13.44% |
| 87 | 7.03E-04 | 13.51% |
| 88 | 7.02E-04 | 13.58% |
| 89 | 7.02E-04 | 13.65% |
| 90 | 7.02E-04 | 13.72% |
| 91 | 7.01E-04 | 13.79% |
| 92 | 7.01E-04 | 13.86% |
| 93 | 7.00E-04 | 13.93% |
| 94 | 7.00E-04 | 14.00% |
| 95 | 6.99E-04 | 14.07% |
| 96 | 6.98E-04 | 14.14% |
| 97 | 6.98E-04 | 14.21% |
| 98 | 6.97E-04 | 14.28% |
| 99 | 6.96E-04 | 14.35% |
| 100 | 6.96E-04 | 14.42% |
