# Supplementary material for: Probabilistic ancestry maps: a method to assess and visualize population substructures in genetics
Source: BMC Bioinformatics. 2019 Mar 7;20:116. doi: 10.1186/s12859-019-2680-1 (PMC6407257; doi:10.1186/s12859-019-2680-1)
Supplement: Supplementary file 12 — 5-fold cross-validated recall for twenty 1000 Genomes Project populations (19 classes) using SVM, PCA or GTM. Recall of optimized models for the following algorithms: SVM 10 PCs = support vector machine classification model using 10 principal components, PCA 8-NN = k-nearest neighbours model based on 2D PCA map (k = 8), GTM 3 or 10 PCs = bayesian classification model based on generative topographic mapping using 3 or 10 principal components. File name: recall_crossvalidation_19classes.html. (HTML 8 kb) [file 12859_2019_2680_MOESM12_ESM.html]

| Ancestry | 1000G code | Population | PCA 8-NN | SVM 10 PCs | GTM 3 PCs | GTM 10 PCs |
| --- | --- | --- | --- | --- | --- | --- |
| EAS | CHB | Han Chinese | 0.17 ± 0.01 | 0.73 ± 0.03 | 0.38 ± 0.06 | 0.62 ± 0.02 |
| EAS | JPT | Japanese | 0.47 ± 0.03 | 0.99 ± 0.00 | 0.98 ± 0.01 | 0.99 ± 0.00 |
| EAS | CHS | Southern Han Chinese | 0.25 ± 0.02 | 0.86 ± 0.03 | 0.46 ± 0.02 | 0.96 ± 0.01 |
| EAS | CDX | Chinese Dai | 0.20 ± 0.02 | 0.05 ± 0.01 | 0.42 ± 0.04 | 0.30 ± 0.07 |
| EAS | KHV | Kinh in Vietnam | 0.58 ± 0.02 | 0.97 ± 0.00 | 0.78 ± 0.04 | 0.92 ± 0.04 |
| EUR | CEU + GBR | Northern and Western European | 0.69 ± 0.02 | 1.00 ± 0.00 | 0.81 ± 0.02 | 0.99 ± 0.01 |
| EUR | TSI | Toscani | 0.51 ± 0.02 | 0.59 ± 0.03 | 0.63 ± 0.03 | 0.39 ± 0.07 |
| EUR | FIN | Finnish | 0.96 ± 0.01 | 0.98 ± 0.01 | 0.98 ± 0.01 | 0.97 ± 0.01 |
| EUR | IBS | Iberian | 0.35 ± 0.03 | 0.97 ± 0.01 | 0.28 ± 0.04 | 0.96 ± 0.02 |
| AFR | YRI | Yoruba in Nigeria | 0.29 ± 0.02 | 1.00 ± 0.00 | 0.11 ± 0.02 | 0.85 ± 0.10 |
| AFR | LWK | Luhya | 0.89 ± 0.01 | 1.00 ± 0.00 | 0.96 ± 0.02 | 1.00 ± 0.00 |
| AFR | GWD | Gambian | 0.28 ± 0.02 | 0.91 ± 0.03 | 0.20 ± 0.02 | 0.64 ± 0.09 |
| AFR | MSL | Mende | 0.19 ± 0.02 | 0.98 ± 0.04 | 0.37 ± 0.03 | 1.00 ± 0.00 |
| AFR | ESN | Esan in Nigeria | 0.22 ± 0.02 | 0.00 ± 0.00 | 0.14 ± 0.05 | 0.22 ± 0.13 |
| AMR | PUR | Puerto Ricans | 0.92 ± 0.01 | 0.78 ± 0.03 | 0.93 ± 0.01 | 0.84 ± 0.06 |
| AMR | CLM | Colombians | 0.62 ± 0.02 | 0.95 ± 0.02 | 0.82 ± 0.02 | 0.86 ± 0.04 |
| AMR | PEL | Peruvians | 0.83 ± 0.01 | 0.95 ± 0.01 | 0.92 ± 0.01 | 0.92 ± 0.01 |
| SAS | PJL | Punjabi | 0.98 ± 0.01 | 0.94 ± 0.01 | 0.95 ± 0.02 | 0.94 ± 0.01 |
| SAS | BEB | Bengali | 0.97 ± 0.00 | 0.99 ± 0.01 | 0.97 ± 0.00 | 0.98 ± 0.01 |
| Overall recall |  |  | 0.55 ± 0.00 | 0.83 ± 0.00 | 0.64 ± 0.00 | 0.81 ± 0.01 |
