# Supplementary material for: Probabilistic ancestry maps: a method to assess and visualize population substructures in genetics
Source: BMC Bioinformatics. 2019 Mar 7;20:116. doi: 10.1186/s12859-019-2680-1 (PMC6407257; doi:10.1186/s12859-019-2680-1)
Supplement: Supplementary file 14 — 5-fold cross-validated recall for five 1000 Genomes Project superpopulations (5 classes). Recall of optimized models for the following algorithms: SVM 10 PCs = support vector machine classification model using 10 principal components, PCA 8-NN = k-nearest neighbours model based on 2D PCA map (k = 8), GTM 3 or 10 PCs = bayesian classification model based on generative topographic mapping using 3 or 10 principal components. File name: recall_crossvalidation_5classes.html. (HTML 3 kb) [file 12859_2019_2680_MOESM14_ESM.html]

| Ancestry | 1000G code | PCA 8-NN | SVM 10 PCs | GTM 3 PCs | GTM 10 PCs |
| --- | --- | --- | --- | --- | --- |
| Africans | AFR | 1.00 ± 0.00 | 1.00 ± 0.00 | 1.00 ± 0.00 | 1.00 ± 0.00 |
| Admixed Americans | AMR | 0.90 ± 0.00 | 1.00 ± 0.00 | 0.99 ± 0.00 | 0.99 ± 0.00 |
| East Asians | EAS | 1.00 ± 0.00 | 1.00 ± 0.00 | 1.00 ± 0.00 | 1.00 ± 0.00 |
| Europeans | EUR | 0.99 ± 0.00 | 1.00 ± 0.00 | 1.00 ± 0.00 | 1.00 ± 0.00 |
| South Asians | SAS | 0.99 ± 0.00 | 1.00 ± 0.00 | 1.00 ± 0.00 | 1.00 ± 0.00 |
| Overall recall |  | 0.98 ± 0.00 | 1.00 ± 0.00 | 1.00 ± 0.00 | 1.00 ± 0.00 |
