# Supplementary figures and images for: Probabilistic ancestry maps: a method to assess and visualize population substructures in genetics
Source: BMC Bioinformatics. 2019 Mar 7;20:116. doi: 10.1186/s12859-019-2680-1 (PMC6407257; doi:10.1186/s12859-019-2680-1)

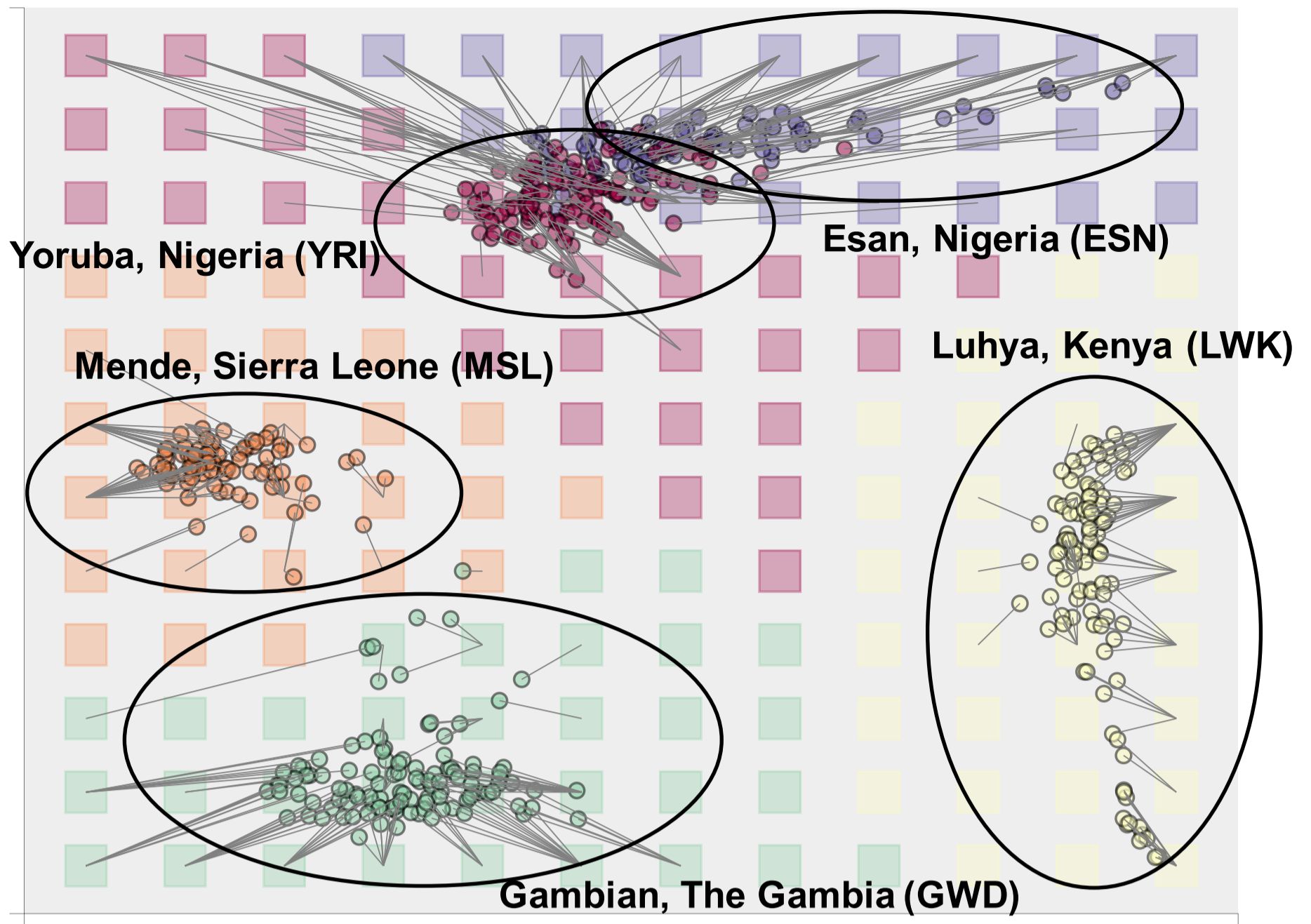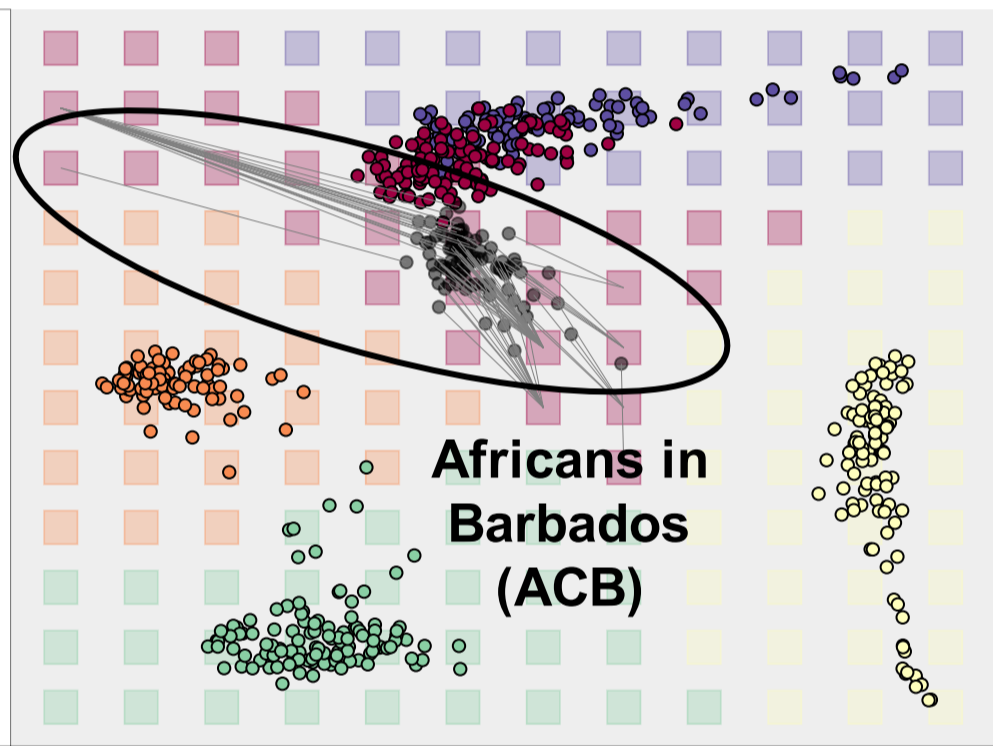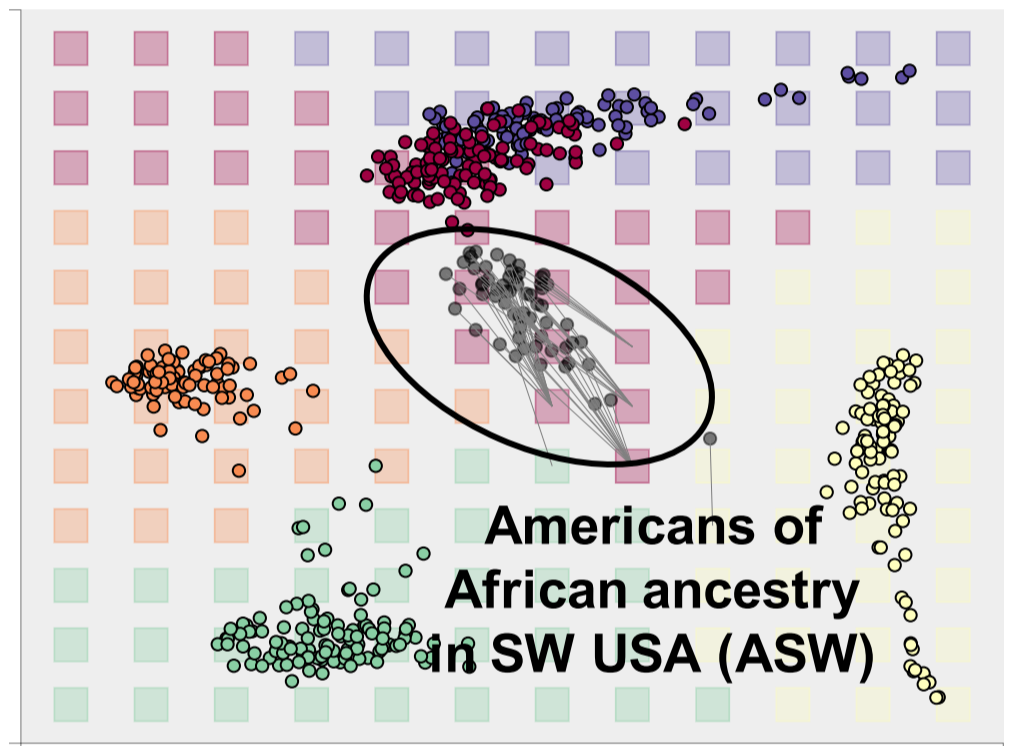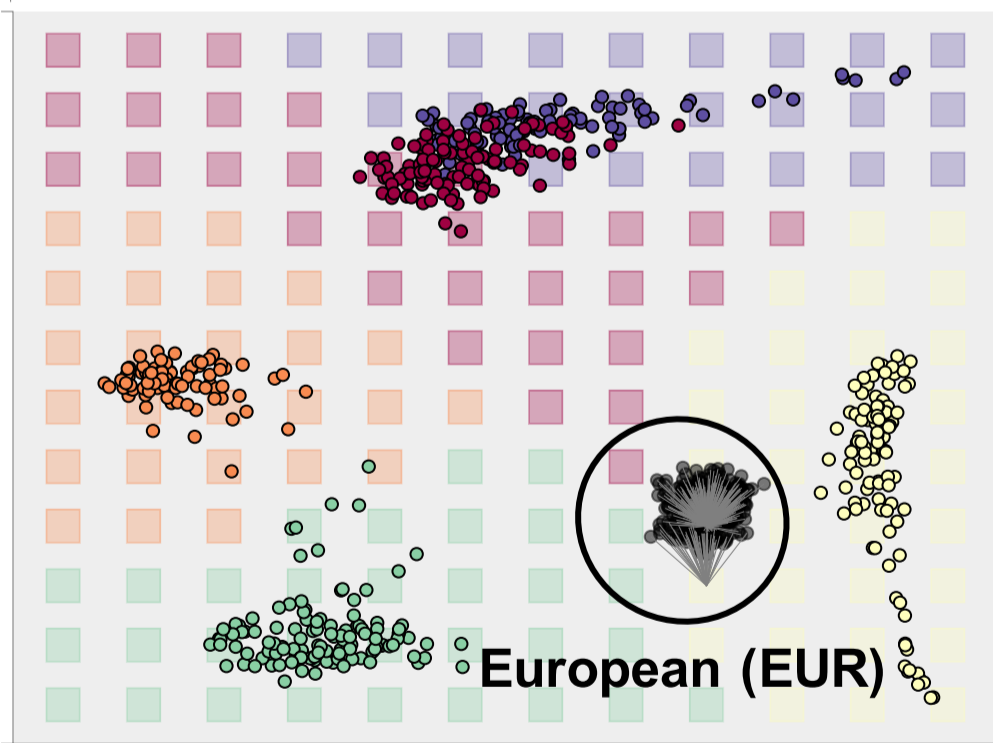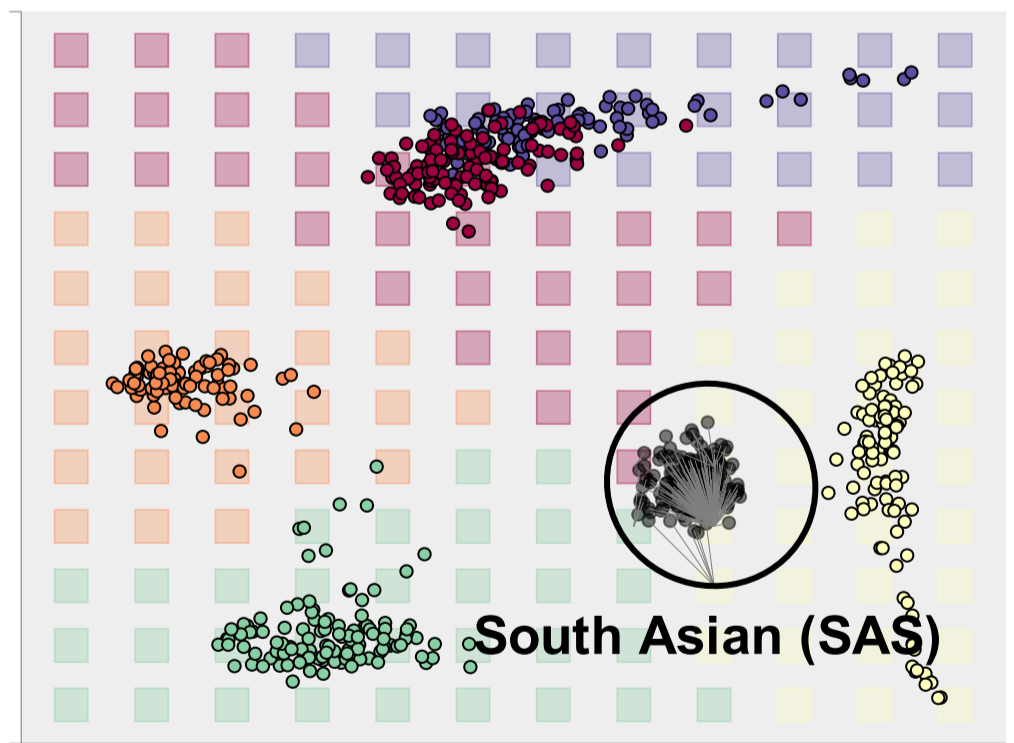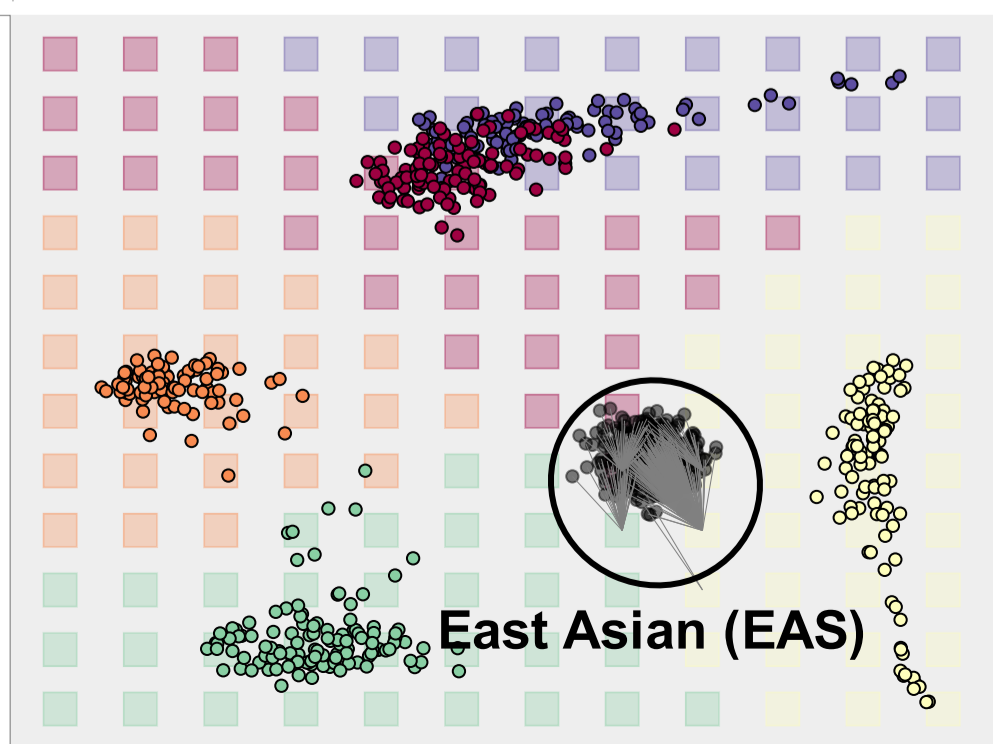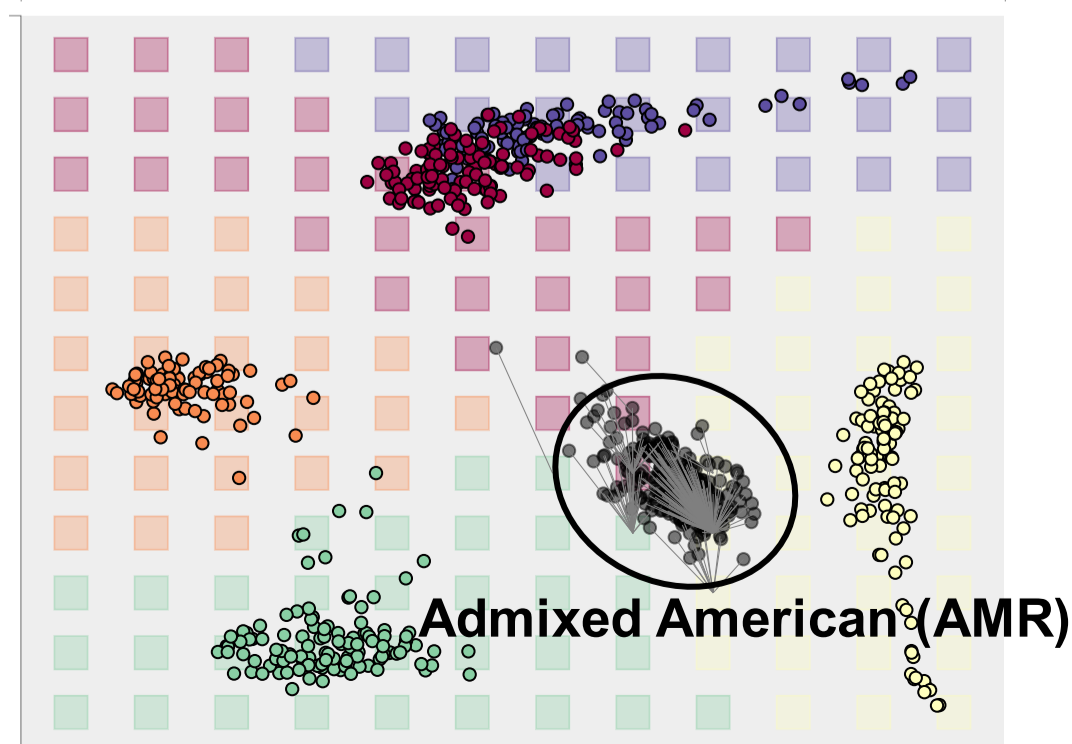

Supplement: Supplementary file 15 — African-only GTM map. Interactive GTM map for AFR superpopulation (1000 Genomes Project), excluding ASW and ACB populations, and projections of following test sets: two African ancestry populations (ASW and ACB), and 1000 Genomes superpopulations (EUR, EAS, AMR, and SAS) on the AFR map).File name: AFR_maps.pdf. (PDF 1414 kb) [file 12859_2019_2680_MOESM15_ESM.pdf]
